# Efficient liquid exfoliation of KP_15_ nanowires aided by Hansen's empirical theory

**DOI:** 10.3762/bjnano.13.69

**Published:** 2022-08-17

**Authors:** Zhaoxuan Huang, Zhikang Jiang, Nan Tian, Disheng Yao, Fei Long, Yanhan Yang, Danmin Liu

**Affiliations:** 1 School of Materials Science and Engineering, Guangxi Key Laboratory of Optical and Electronic Materials and Devices, Collaborative Innovation Center for Exploration of Nonferrous Metal Deposits and Efficient Utilization of Resources, Guilin University of Technology, Guilin 541004, China; 2 School of Science, Xi’an University of Posts and Telecommunications, Xi’an 710121, China; 3 Institute of Microstructure and Property of Advanced Materials, Beijing University of Technology, Beijing, China

**Keywords:** Hansen's empirical theory, KP_15_, liquid exfoliation, nanodevices, nanowires, Raman, semiconductors

## Abstract

The KP_15_ nanowires with one-dimensional properties has a defect-free surface, high anisotropy, and carrier mobility which is desirable for the development of novel nanodevices. However, the preparation of nanoscale KP_15_ is still inefficient. In this work, the Hansen solubility parameters of KP_15_ were first obtained. Based on the Hansen's empirical theory, the concentration of liquid-exfoliated KP_15_ nanowires was improved to 0.0458 mg·mL^−1^ by a solution containing 50% water and 50% acetone. Approximately 79% of the KP_15_ nanowires had a thickness value below 50 nm and 60.9% of them had a width value below 100 nm. The thinnest KP_15_ nanowires reached 5.1 nm and had smooth boundaries. Meanwhile, strong temperature-dependent Raman response in exfoliated KP_15_ nanowires has been observed, which indicates a strong phonon–phonon coupling in those nanowires. This is helpful for non-invasive temperature measurements of KP_15_ nanodevices.

## Introduction

Low-dimensional materials have drawn significant attention in recent years. So far, not only new composite materials with excellent properties have been obtained by the synthesis of different materials, but also low-dimensional materials with different properties than those of bulk materials have been synthesized by physical and chemical methods. For instance, Bingjun Yang used one-dimensional graphene nanoscroll-wrapped MnO nanoparticles as anode materials to promote the rapid diffusion and electron transfer of lithium, and Rongjun Zhao prepared *n*-butanol gas sensors with one-dimensional In_2_O_3_ nanorods [1–2]. Different from 2D materials, 1D materials generally have a chain-like crystal structure and are easily exfoliated due to a weak interaction between these chains [3–4]. Therefore, those 1D materials have defect-free surfaces, high anisotropy, and carrier mobility. For example, TiS_3_ nanowires obtained by mechanical stripping have a large carrier mobility of about 10000 cm^2^·V^−1^·s^−1^ [5–7]. Fibrous phosphorus is also a new one-dimensional material with high carrier mobility (308 cm^2^·V^−1^·s^−1^) and rapid response time [8–10]. These one-dimensional materials are ideal for photovoltaic and photocatalytic applications.

The KP_15_ is considered to be a novel low-dimensional material with layered structure, high hole carrier mobility (1000 cm^2^·V^−1^·s^−1^), and highly anisotropic properties [11]. The photodetectors prepared with KP_15_ have a fast response time and are ideal materials for photovoltaic applications [12]. Based on our previous studies, KP_15_ is also a one-dimensional material with a defect-free surface [13–14]. This is beneficial for the development of high-performance nanodevices. Searching effective synthesis routes for nanoscale KP_15_ has become an urgent issue. Liquid-phase exfoliation is one of the most straightforward methods to prepare low-dimensional materials at a low cost and with simple processes and high flexibility. In this case, the surface of bulk materials is peeled off or corroded by physical or chemical reactions in a liquid medium, and finally low-dimensional materials are obtained. Based on the Hansen's empirical theory, the exfoliation efficiency of low-dimensional materials can be improved by adjusting the composition and type of solutions used in the liquid-phase exfoliation [15–17]. This theory has been successfully used for improving the exfoliation efficiency in several low-dimensional materials, such as carbon, graphene, metal oxides, and fibrous phosphorus. [18].

In a previous study, we exfoliated KP_15_ in alcohol; however, this method was still inefficient [13]. Herein, the Hansen's empirical theory was firstly introduced to improve the liquid-phase exfoliation efficiency of KP_15_ nanowires. In addition, Hansen solubility parameters (HSPs) for KP_15_ were also obtained in this work. By using a solution containing 50% water and 50% acetone, the exfoliation efficiency of KP_15_ was effectively improved. Our results show that 79% of the KP_15_ nanowires had thickness values below 50 nm and 60.9% of these nanowires had width values below 100 nm. The thinnest KP_15_ nanowires reached 5.1 nm and had smooth boundaries. Meanwhile, a strong temperature-dependent Raman response was found in exfoliated KP_15_ nanowires. This indicates a strong phonon–phonon coupling in KP_15_ nanowires, which favors non-invasive temperature measurements of KP_15_ nanodevices.

## Methods

### Synthesis of KP_15_ bulks

The KP_15_ bulks were prepared by the gas-phase transfer method. High-purity red phosphorus (1.370 g, 99.9999%) and metallic potassium (0.130 g, 97%) were mixed in a quartz tube. The temperature gradient in the quartz tube was 650 °C/400 °C and the heat treatment time was 12 h. After annealed, dark-red KP_15_ bulks were finally obtained.

### Liquid exfoliation

For the liquid-exfoliation process, 1 mg of KP_15_ was mixed in 20 mL of solvent and ultrasonically processed at a power of 80 W in an ice bath for 6 h, followed by centrifugation at 2000 rpm for 20 min. For the samples with predetermined concentration, centrifugation was not used.

### Measurement equipment

UV−visible spectrophotometry was performed by using a Shimadzu UV-3101PC system. Atomic force microscopy (AFM) tests were performed in a Multimode 8 system. The Raman tests were performed on a WITec alpha300 RA confocal Raman microscopy system. For the Raman tests, KP_15_ samples were spun on SiO_2_(300 nm)/Si substrates. The excitation wavelength used was 532 nm, the spot size was approx. 1 μm, and the laser power was kept below 20 μW. For low-temperature Raman measurements, a Linkam THMS600 cryostat cooled by liquid nitrogen was used to control the temperature. To prevent sample drift, SiO_2_ (300 nm)/Si substrates with tested KP_15_ samples were attached by fixtures to the Linkam THMS600 cryostat.

## Results and Discussion

**Figure 1:**
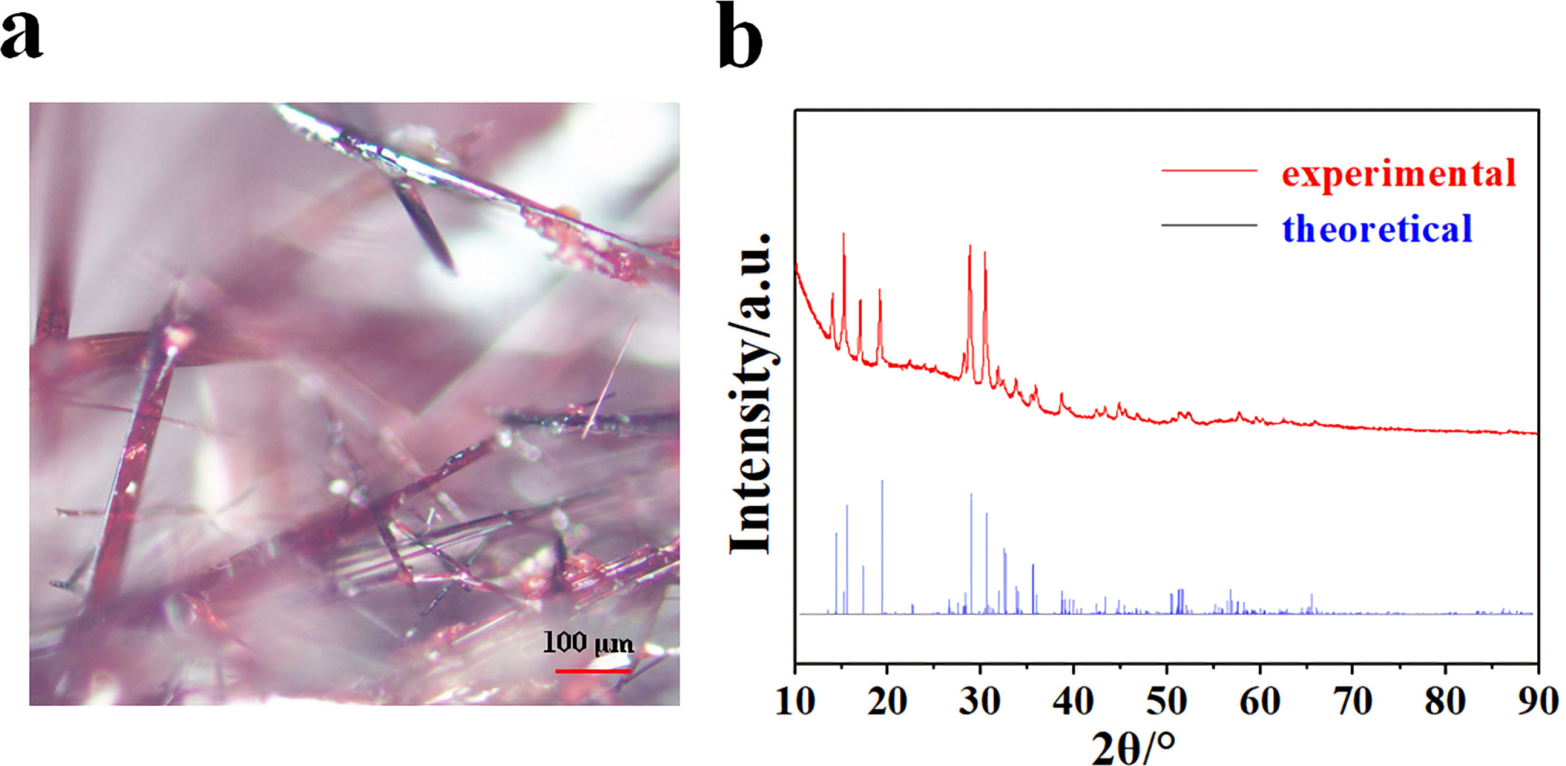
(a) Optical microscopy result of KP_15_ bulks. (b) XRD results of KP_15_ bulks.

KP_15_ bulks, prepared by the gas-phase-transfer method, had a flat and smooth surface shown in Figure 1a. The X-ray diffraction patterns of the synthesized KP_15_ were both theoretically calculated and experimentally measured. The consistency between the two patterns shows that there is no impurity phase (Figure 1b), which confirms an excellent crystallization quality of the KP_15_ bulks.

### Measurement of the absorption coefficient and the Hansen solubility parameters for KP_15_

According to the Hansen’s theory [19], the dispersed concentration *C* of a KP_15_ dispersion prepared by liquid exfoliation can be expressed by Equation 1 as follows.


[1] $$\begin{array}{l} 1/C\propto \tau ={\left(\delta_{\text{A,D}}-\delta_{\text{B,D}}\right)}^{2}\\ \;+{\left(\delta_{\text{A,P}}-\delta_{\text{B,P}}\right)}^{2}/4+{\left(\delta_{\text{A,H}}-\delta_{\text{B,H}}\right)}^{2}/4, \end{array}$$

where δ_D_ is the intermolecular dispersion force, δ_H_ is the intermolecular hydrogen bond; δ_P_ is the intermolecular polar force; δ_A,D_, δ_A,P_, δ_A,H_ are the Hansen solubility parameters (HSPs) of the solute; and δ_B,D_, δ_B,P_, δ_B,H_ are the HSPs of the solvent. Therefore, to get a high concentration of KP_15_ in dispersion, the HSPs of the solvent for the exfoliation of KP_15_ should be close to those of KP_15_. A weighted average method was used to calculate the HSPs of KP_15_. The concentration of KP_15_ was used as a weight factor for each suspension. This way, the HSPs of KP_15_ can be expressed according to Equation 2 [19].


[2] $$\delta_{i}=\frac{\sum C\delta_{i,\text{sol}}}{\sum C},$$

where δ*_i_*_,sol_ are the HSPs of the solvent and *C* is the concentration of the KP_15_ dispersions. The Lambert–Beer law (Equation 3) was then used to measure the concentration of the KP_15_ dispersions:


[3] A=KbC,

where *A* is the absorbance, *K* is the absorption coefficient of the material, *b* is the absorbing layer thickness (which in this work is the width of the cuvette, i.e., 1 cm), and *C* is the concentration of the KP_15_ dispersions. The absorbance *A* and the absorption coefficient *K* are related to the wavelength of the incident light. To determine *A* and *K*, it is necessary to choose a specific incident wavelength. The bandgap of bulk KP_15_ is approx. 1.75 eV [20]. However, according to our previous study, with thickness reduction of the KP_15_ nanowires, a surface-state luminescence at 693 nm gradually dominates in the KP_15_ nanowire [14]. This could affect light absorption properties of KP_15_ due to its decreased size.

**Figure 2:**
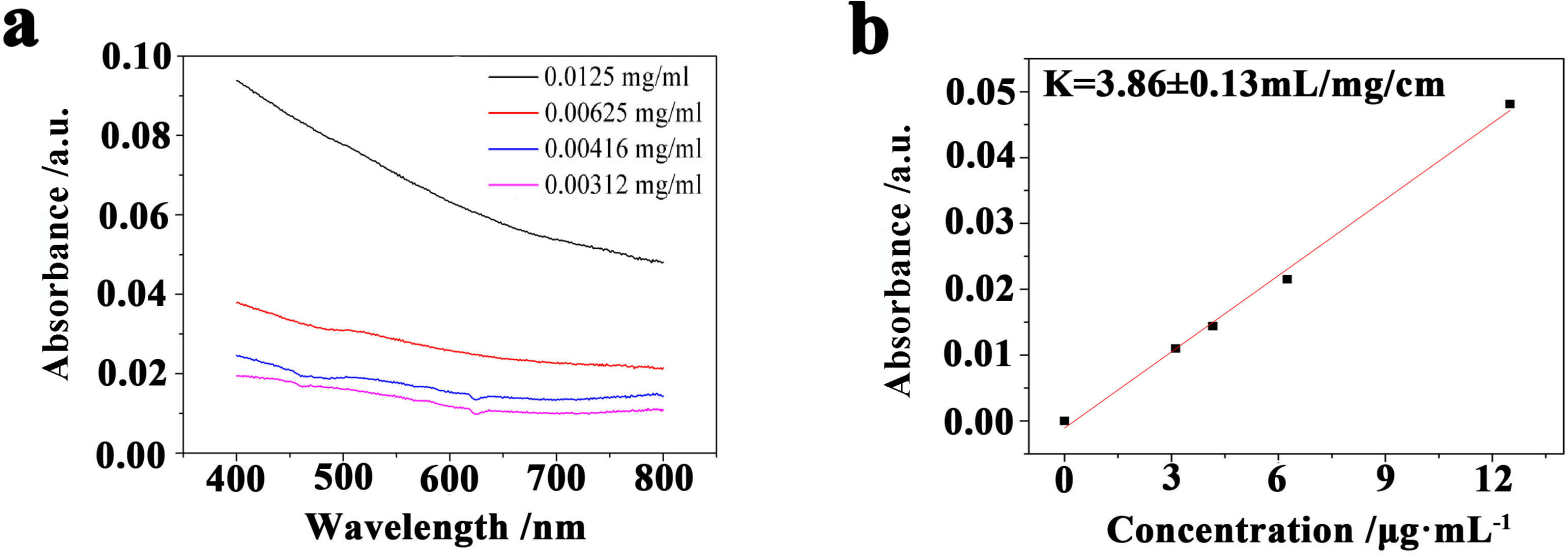
Absorbance of predetermined KP_15_ dispersions exfoliated in butyrolactone. (a) Absorbance of different concentrations of predetermined KP_15_ dispersions exfoliated in butyrolactone. (b) Absorbance (800 nm) as a function of concentration of predetermined KP_15_ dispersions. The absorption coefficient (800 nm) is 3.86 ± 0.13 mL·mg^−1^·cm^−1^.

To avoid the generation of concentration error caused by the absorbance influence of the surface state, a wavelength (800 nm) which is far away from the bandgap of KP_15_ bulk and surface state in the KP_15_ nanowires was chosen. Some dispersions for which we predetermined the concentration were prepared to fit and determine the absorption coefficient *K*. Solutions of five different concentrations of KP_15_ dispersions in butyrolactone were prepared by liquid exfoliation with a predetermined concentration. UV−visible absorption spectra results are shown in Figure 2. The concentration linearly varies with absorbance. The slope of this fitted linear equation is 3.86 ± 0.13. This means that the absorption coefficient of KP_15_ is 3.86 ± 0.13 mL·mg^−1^·cm^−1^.

We selected 20 common solvents, including benzyl benzoate, toluene, ethyl acetate, acetone, alcohol, butyrolactone, *N*,*N*'-dimethylpropyleneurea, bromobenzene, cyclopentanone, *N*-dodecyl-2-pyrrolidone, glycol, vinyl acetate, hexane, isopropyl alcohol, *N*,*N*-dimethylformamide, *O*-phthalic dimethyl ester, dimethyl sulfoxide, *N*-methylpyrrolidone, water, and cyclohexanone. The HSPs of those solvents are listed in Table 1.

**Table 1:** Hansen parameters for the solvents [21].

solvent	δ_D_ (MPa^1/2^)	δ_P_ (MPa^1/2^)	δ_H_ (MPa^1/2^)

benzyl benzoate	20	5.1	5.2
toluene	18	1.4	2
ethyl acetate	15.8	5.3	7.2
acetone	15.5	10.4	7
alcohol	18.1	17.1	16.9
butyrolactone	18	16.6	7.4
*N*,*N*'-dimethylpropyleneurea	17.8	9.5	9.3
bromobenzene	19.2	5.5	4.1
cyclopentanone	17.9	11.9	5.2
*N*-dodecyl-2-pyrrolidone	17.5	4.1	3.2
glycol	17	11	26
vinyl acetate	16	7.2	5.9
hexane	14.9	0	0
isopropyl alcohol	15.8	6.1	16.4
*N*,*N*-dimethylformamide	17.4	13.7	11.3
*O*-phthalic dimethyl ester	18.6	10.8	4.9
dimethyl sulfoxide	18.4	16.4	10.2
*N*-methylpyrrolidone	18	12.3	7.2
water	15.8	8.8	19.4
cyclohexanone	17.8	8.4	5.1

**Figure 3:**
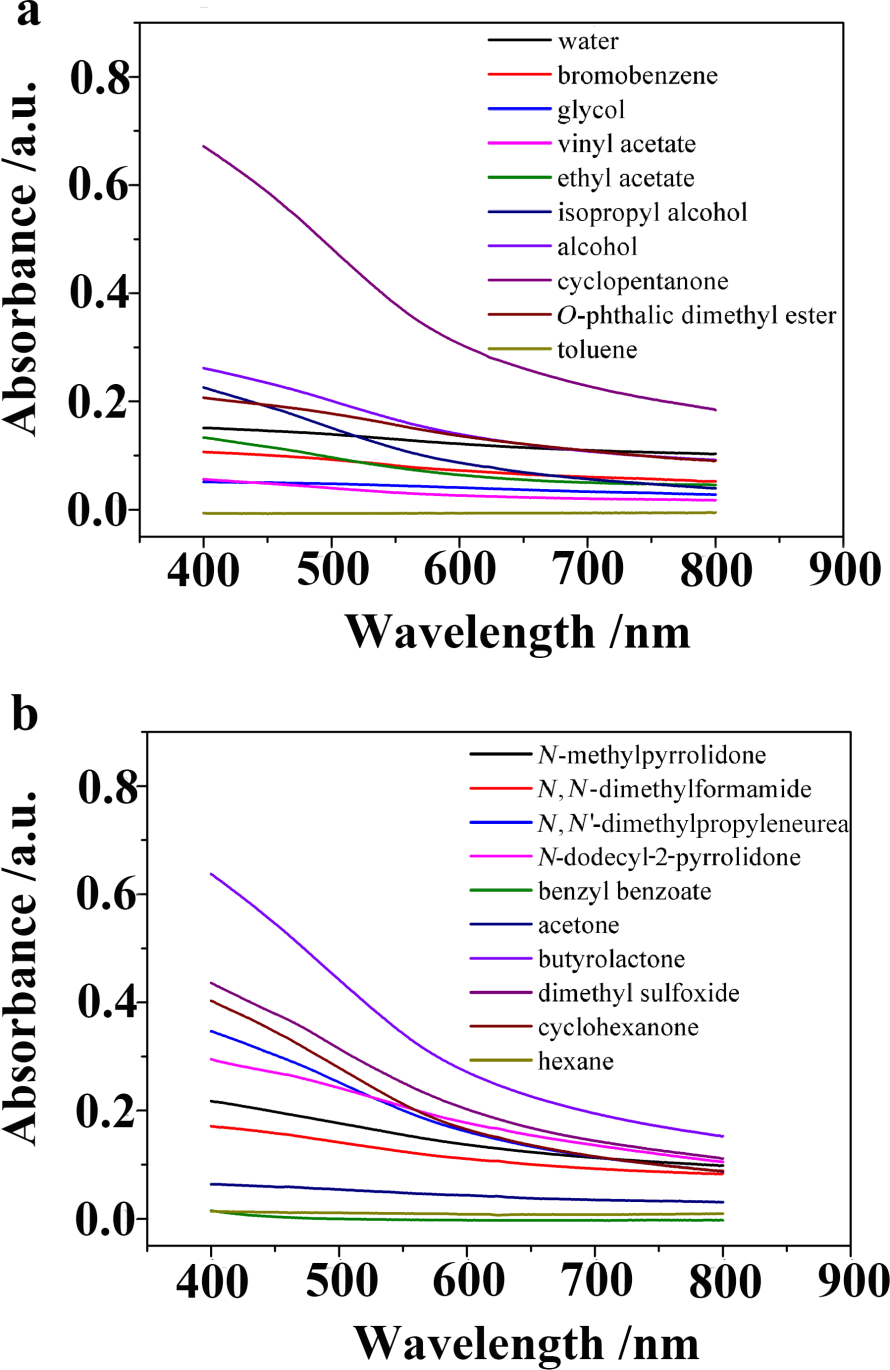
UV−visible spectrum of the KP_15_ dispersions using various solvents.

**Figure 4:**
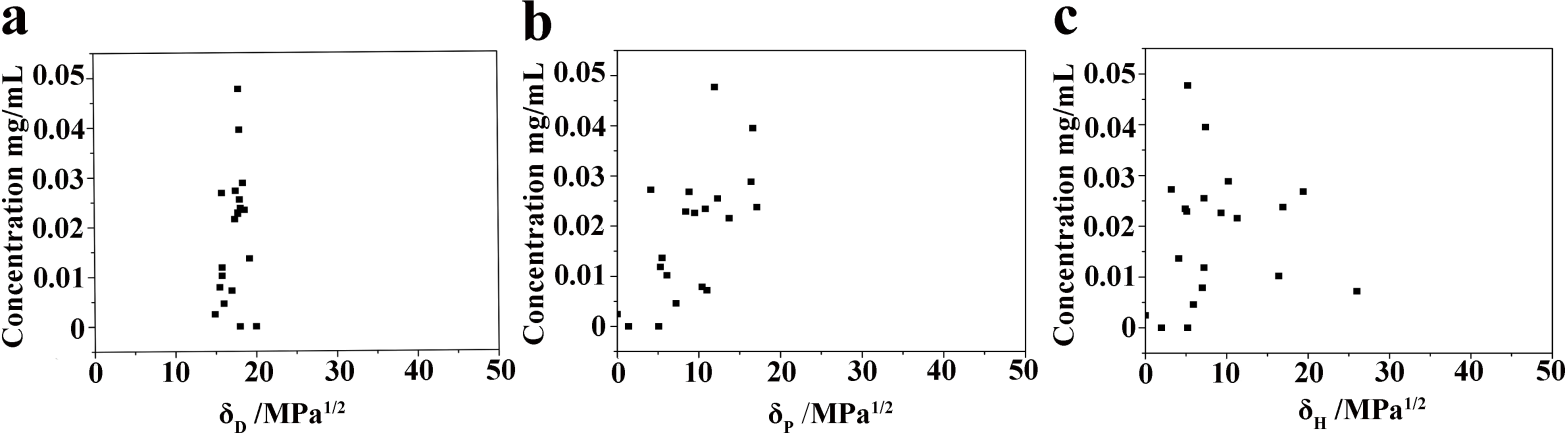
Concentration of KP_15_ dispersions as a function of the Hansen parameters. (a) Concentration of KP_15_ dispersions as a function of δ_D_. (b) Concentration of KP_15_ dispersions as a function of δ_P_. (c) Concentration of KP_15_ dispersions as a function of δ_H_.

**Figure 5:**
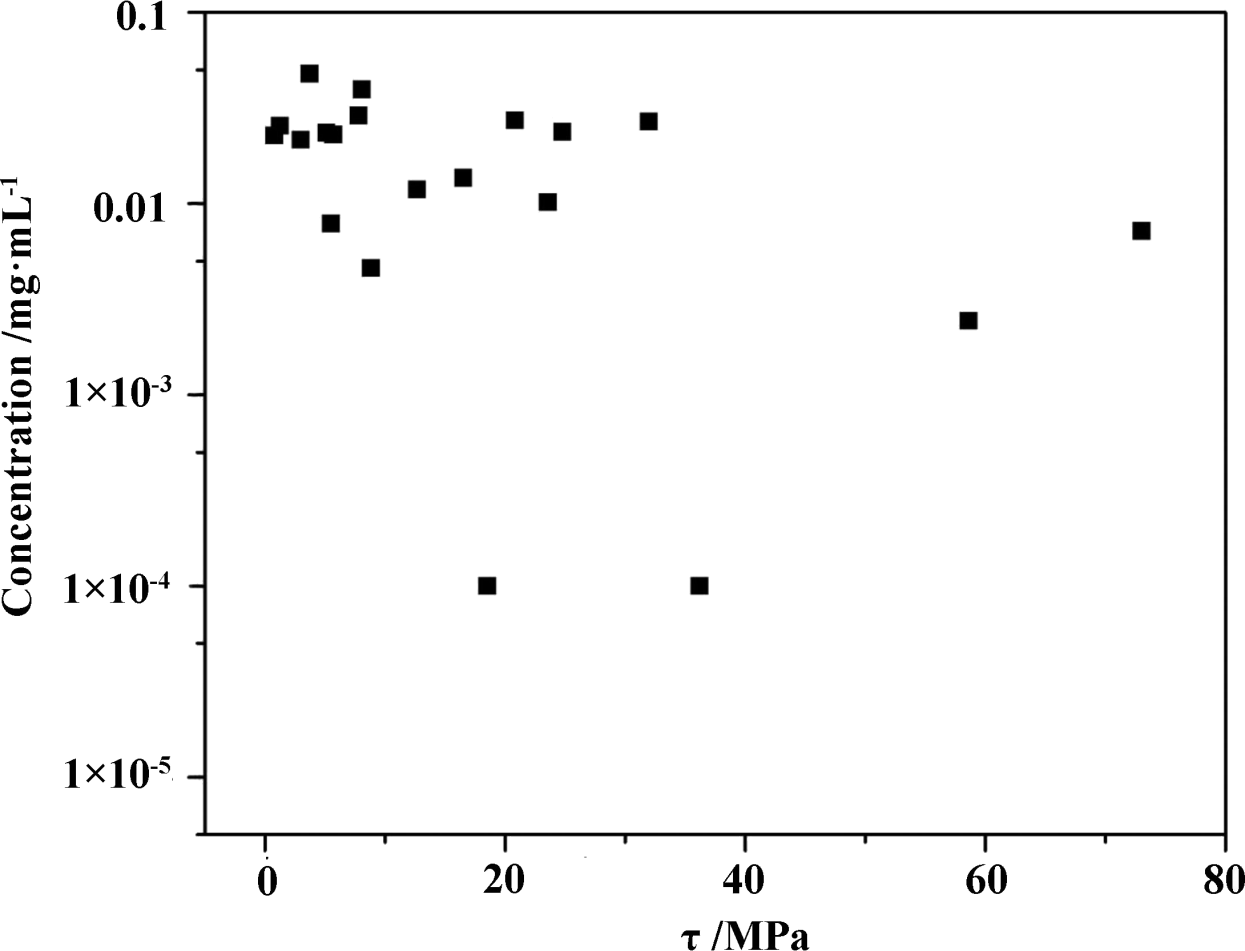
Concentration of KP_15_ dispersions as a function of τ, τ = [(δ_A,D_ − δ_B,D_)^2^ + (δ_A,P_ − δ_B,P_)^2^/4 + (δ_A,H_ − δ_B,H_)^2^/4].

Figure 3 exhibits the concentrations of KP_15_ dispersions exfoliated in different solvents. Cyclopentanone and butyrolactone were more suitable than the other solvents to exfoliate KP_15_. Figure 4 shows the relationship between the HSPs of different solvents and the concentration of the KP_15_ suspension. Based on Equation 2, the HSPs of KP_15_ were δ_D_ = 17.60 MPa^1/2^, δ_P_ = 11.19 MPa^1/2^, and δ_H_ = 8.95 MPa^1/2^. As long as the difference between the HSPs of KP_15_ and the HSPs of a given solvent is reduced, τ can be reduced with an improved exfoliation efficiency. Figure 5 shows the concentration of KP_15_ dispersions as a function of τ. When τ tends to zero, the concentration of the KP_15_ dispersion reaches the maximum value, which corresponds to the results of the aforementioned equation.

### Liquid exfoliation of one-dimensional KP_15_

The HSPs obtained for KP_15_ were δ_D_ = 17.60 MPa^1/2^, δ_P_ = 11.19 MPa^1/2^, and δ_H_ = 8.95 MPa^1/2^. We chose a mixed solution containing water and acetone to exfoliate KP_15_. The HSPs of water were δ_D_ = 15.8 MPa^1/2^, δ_P_ = 8.8 MPa^1/2^, and δ_H_ = 19.4 MPa^1/2^. The HSPs of acetone were δ_D_ = 15.5 MPa^1/2^, δ_P_ = 10.4 MPa^1/2^, and δ_H_ = 7.0 MPa^1/2^. The HSP range of a mixed solution of water and acetone can cover the HSPs of KP_15_, however, both of them can be easily removed. The HSPs (δ*_i_*) in a mixed solution containing water and acetone can be expressed by Equation 4.


**Figure 6:**
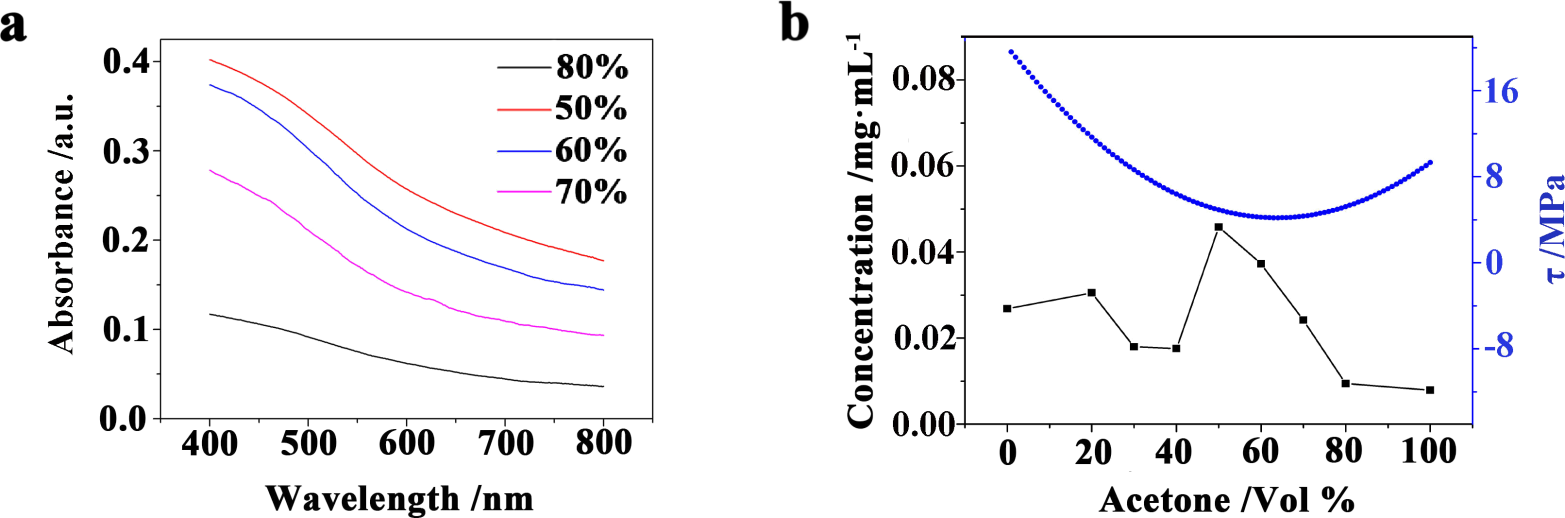
Results of KP_15_ dispersions exfoliated in acetone/water mixtures. (a) Absorbance of KP_15_ dispersions exfoliated in acetone/water mixtures with different acetone volume fractions. (b) KP_15_ suspension concentration and the calculated τ as a function of the acetone volume fraction.

[4] $$\delta_{i}=\sum \phi_{i,\text{comp}}\delta_{i,\text{comp}},$$

where ϕ*_i_*_,comp_ is the volume fraction of the corresponding solvent and δ*_i_*_,comp_ is the HSPs of the solvent. The concentration of the KP_15_ dispersion can be measured by the Lambert–Beer law (Equation 3). As shown in Figure 6a, by tuning the volume fraction of acetone in the mixed solution, the HSPs of the mixed solution can be close to those of KP_15_, and the exfoliation efficiency can be clearly improved. The concentration values of the KP_15_ suspension in the solutions were 0.0268 mg·mL^−1^ (exfoliated in deionized water), 0.0079 mg·mL^−1^ (acetone), and 0.0236 mg·mL^−1^ (alcohol), respectively [13]. When the solvent mixture with a 50% volume fraction of acetone is used for stripping, the concentration of the KP_15_ dispersion finally increases to 0.0458 mg·mL^−1^. At this point, the parameter τ is close to the minimum value.

**Figure 7:**
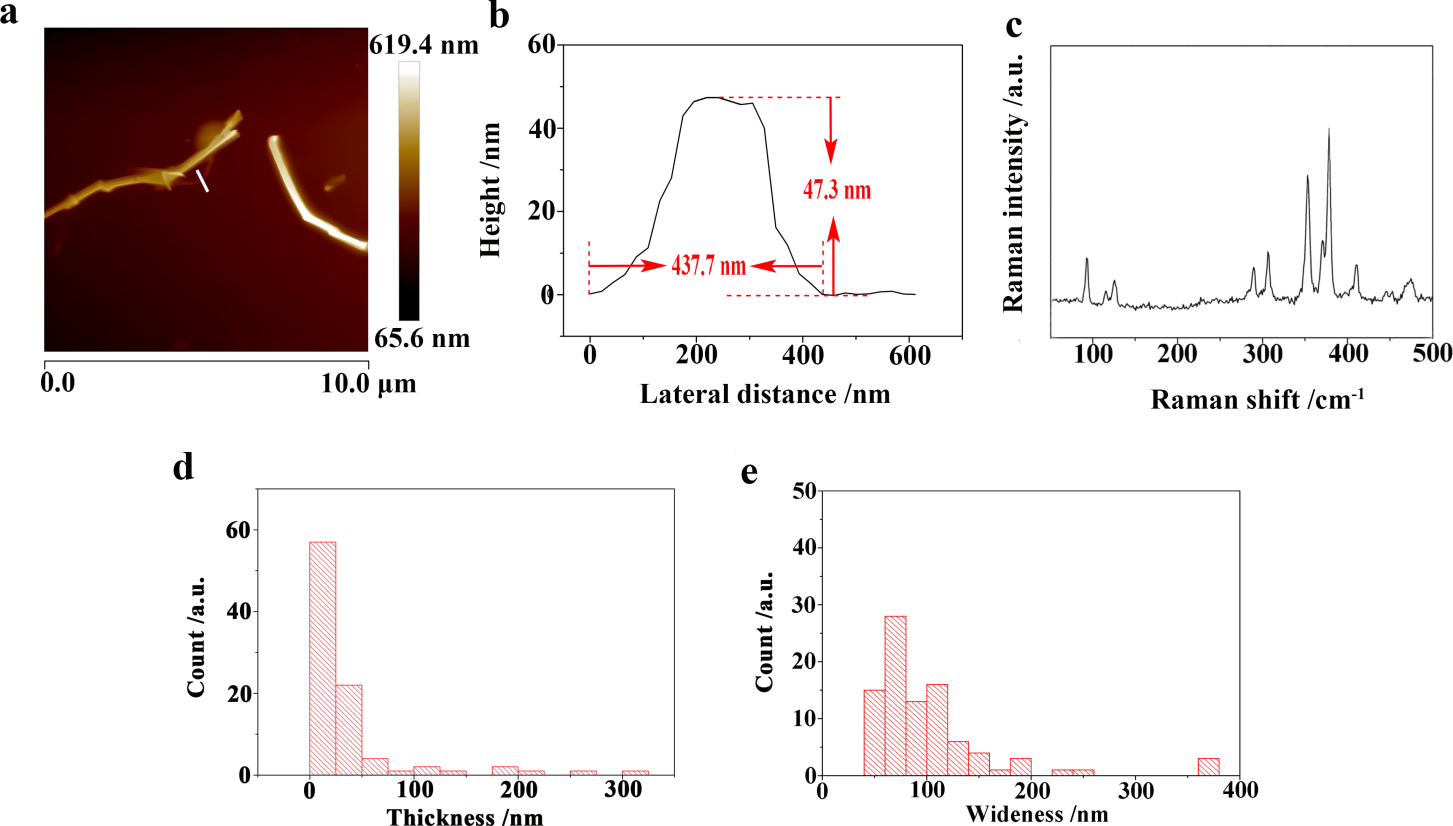
(a) Height distribution of KP_15_ nanowires after liquid exfoliation. (b) Cross sections of KP_15_ nanowires after liquid exfoliation. (c) Raman spectra of KP_15_ nanowires after liquid exfoliation. (d) Thickness histograms of KP_15_ nanowires after liquid exfoliation. (e) Width histograms of KP_15_ nanowires after liquid exfoliation.

**Figure 8:**
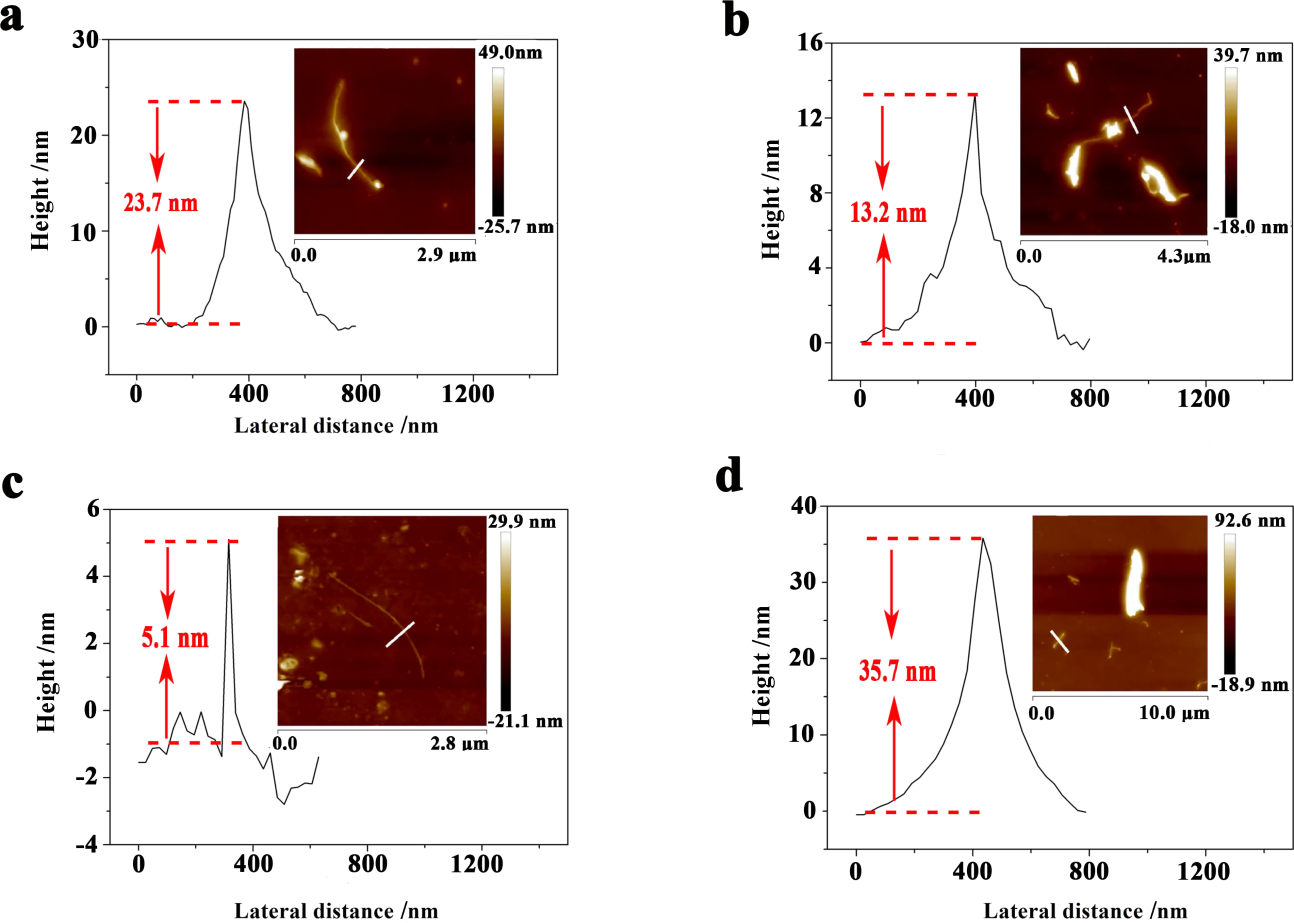
Sizes of exfoliated KP_15_ nanowires. (a) Cross section of the KP_15_ nanowire marked in the upper right corner inset image. (b) Cross section of the KP_15_ nanowire marked in the upper right corner inset image. (c) Cross section of the KP_15_ nanowire marked in the upper right corner inset image. (d) Cross section of the KP_15_ nanowire marked in the upper right corner inset image.

The Raman result for the KP_15_ nanowires exfoliated in water−acetone mixed solution is shown in Figure 7c. At least 11 distinguishable Raman peaks located at 476.6, 453.0, 408.8, 378.3, 368.4, 354.1, 303.7, 288.5, 126.1, 114.1, and 90.7 cm^−1^ were seen and those Raman results were similar to the Raman modes of mechanically exfoliated KP_15_ [11]. As shown in Figure 7d, Figure 7e, and Figure 8, the thinnest KP_15_ nanowires obtained by liquid exfoliation could reach 5.1 nm and had smooth boundaries. The thicknesses of 79% of the liquid-exfoliated KP_15_ nanowires were below 50 nm; the widths of 60.9% of the KP_15_ nanowires were below 100 nm. The sizes of the obtained KP_15_ nanowires were much smaller than those obtained in our previous studies [13]. Meanwhile, a strong temperature-dependent Raman response in exfoliated KP_15_ nanowires has been observed. That may help with non-invasive temperature measurements of KP_15_ nanodevices (details are demonstrated in Supporting Information File 1).

## Conclusion

In summary, based on the Hansen's empirical theory, the liquid phase exfoliation efficiency of KP_15_ nanowires has been improved. The HSPs of KP_15_ were calculated to be δ_D_ = 17.60 MPa^1/2^, δ_P_ = 11.19 MPa^1/2^, and δ_H_ = 8.95 MPa^1/2^. In addition, based on the Hansen's empirical theory, the exfoliation efficiency was improved by adjusting the ratio of water and acetone. When the mixed solvents had the smallest τ, the thicknesses of 79% of liquid-exfoliated KP_15_ nanowires were below 50 nm and the widths of 60.9% of KP_15_ nanowires were below 100 nm. Meanwhile, a strong temperature-dependent Raman response has been found in exfoliated KP_15_, which may help with non-invasive temperature measurements of KP_15_ nanodevices.

## Supporting Information

File 1 Strong temperature-dependent Raman response of exfoliated KP_15_.
