# Supplementary material for: Efficient liquid exfoliation of KP15 nanowires aided by Hansen's empirical theory
Source: Beilstein J Nanotechnol. 2022 Aug 17;13:788–95. doi: 10.3762/bjnano.13.69 (PMC9443416; doi:10.3762/bjnano.13.69)
Supplement: File 1 — Strong temperature-dependent Raman response of exfoliated KP15. [file Beilstein_J_Nanotechnol-13-788-s001.pdf]

BEILSTEIN JOURNAL OF NANOTECHNOLOGY

Supporting Information

for

Efficient liquid exfoliation of KP_{15} nanowires aided by Hansen's empirical theory

Zhaoxuan Huang, Zhikang Jiang, Nan Tian, Disheng Yao, Fei Long, Yanhan Yang and Danmin Liu

Beilstein J. Nanotechnol. **2022,** *13,* 788–795. doi:10.3762/bjnano.13.69

Strong temperature-dependent Raman response of exfoliated KP_{15}

License and Terms: This is a supporting information file under the terms of the Creative Commons Attribution License (https://creativecommons.org/licenses/by/4.0). Please note that the reuse, redistribution and reproduction in particular requires that the author(s) and source are credited and that individual graphics may be subject to special legal provisions.
The license is subject to the *Beilstein Journal of Nanotechnology* terms and conditions: (https://www.beilstein-journals.org/bjnano/terms)

The KP_{15} nanowires with a defect-free surface and high carrier mobility are suitable for the development of novel electric nanodevices. The vibrational properties of KP_{15} nanowires can play an important role in the electronic performance of nanodevices [1]. Here we found that exfoliated KP_{15} nanowires had a strong temperature-dependent Raman response. Due to the resolution of the Raman system at a low-temperature mode, a KP_{15} nanowire with a thickness of 80.9 nm and a width of 139.1 nm was chosen for the temperature-dependent Raman test (Figure S1a and Figure S1b). When the laser power values were 20 and 80 μw, respectively, the positions of those Raman peaks were the same (Figure S1c). This means that when the laser power was below 80 μw, the laser heating did not significantly increase the temperature of the KP_{15} nanowires. Here, for low-temperature Raman measurements, the laser power was kept below 20 μW. Figure S1d shows Raman spectra of the tested KP_{15} nanowire under different temperatures. The space group of KP_{15} is $P\overline{1}$, so all the Raman peaks in KP_{15} are in the A_g mode. There were seven Raman peaks with a high signal-to-noise ratio located at 90.6, 126.1, 354.2, 369.6, 378.7, 453.9, and 476.2 cm^{-1}. These peaks were named as No. 1, 4, 7, 8, 9, 11, and 12 Raman peaks for an easy identification in Figure S1d. As the temperature increased, all the Raman peaks of the liquid-exfoliated KP_{15} nanowire shifted towards lower wave numbers (Figure S1). The vibration frequencies of those Raman peaks have a linear relationship with temperature (Figure S2). The peak position and temperature data can be fitted by the following the Equation S1.

$$\omega = \omega_0 + \chi T, \qquad \text{(S1)}$$

where ω_o is the vibration frequency of the corresponding Raman mode at absolute zero, χ is the first-order temperature coefficient of the Raman mode, and ω is the vibration frequency of the Raman mode at T [2]. The second-order term was not considered because it usually appeared at high temperature. The temperature evolutions of those Raman modes in the tested KP_{15} are shown in Figure S2. For peak 1, χ_1=−0.00942 $cm^{-1}K^{-1}$; for peak 4, χ_4=−0.01756 $cm^{-1}K^{-1}$; for peak7, χ_7=−0.02118 $cm^{-1}K^{-1}$; for peak 8, χ_8=−0.02065 $cm^{-1}K^{-1}$; for peak 9, χ_9=−0.01327 $cm^{-1}K^{-1}$; for peak 11, χ_{11}=−0.01892 $cm^{-1}K^{-1}$; and for peak 12, χ_{12}=−0.01724 $cm^{-1}K^{-1}$. The highest temperature coefficient (χ_7=−0.02118 $cm^{-1}K^{-1}$) in

KP_{15} nanowire Raman modes is near to the highest value of black phosphorus (−0.028 $cm^{-1}K^{-1}$) and is higher than the highest value of MoS_2 (−0.0132 $cm^{-1}K^{-1}$) and graphene (−0.016 $cm^{-1}K^{-1}$) [2-4].

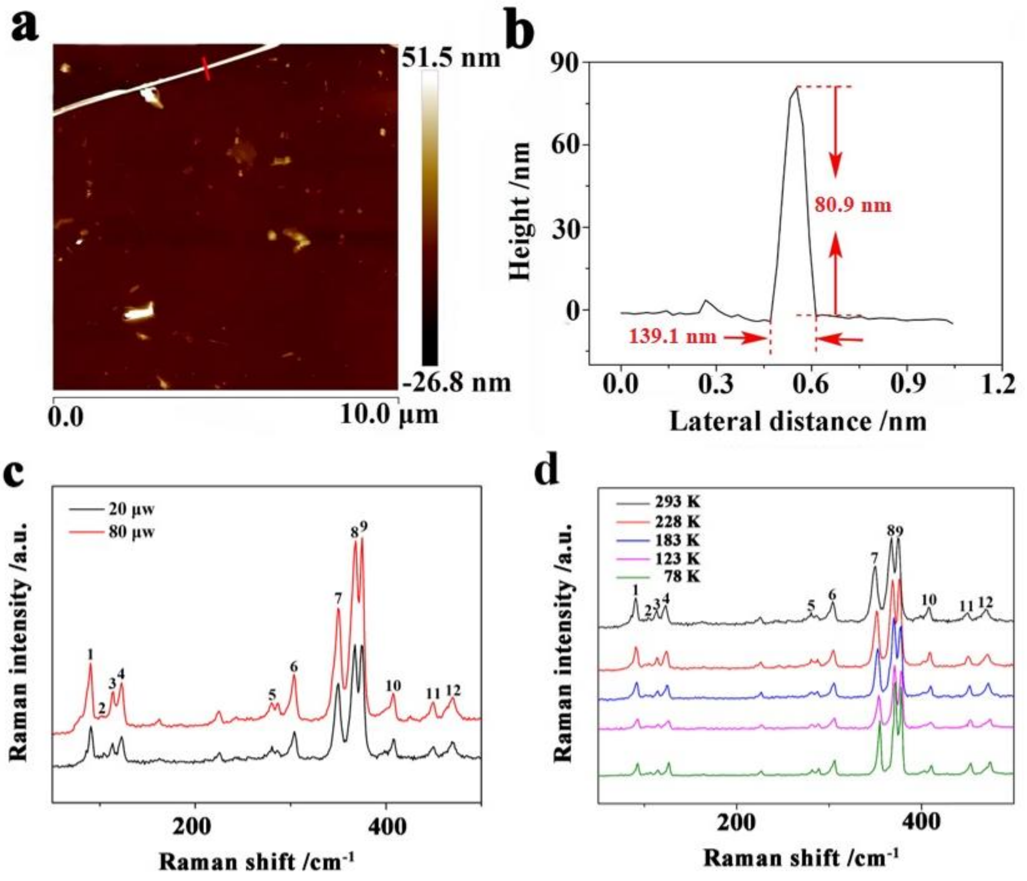

Figure S1: The size of the tested KP_{15} nanowire and its Raman results at different temperatures. (a) Optical result of the tested KP_{15} nanowire. (b) AFM result of the tested KP_{15} nanowire marked in Figure S1a. (c) Raman results of the tested KP_{15} nanowire marked in Figure S1a with different laser power values at room temperature. (d) Raman results of the tested KP_{15} nanowire marked in Figure S1a at different temperatures.

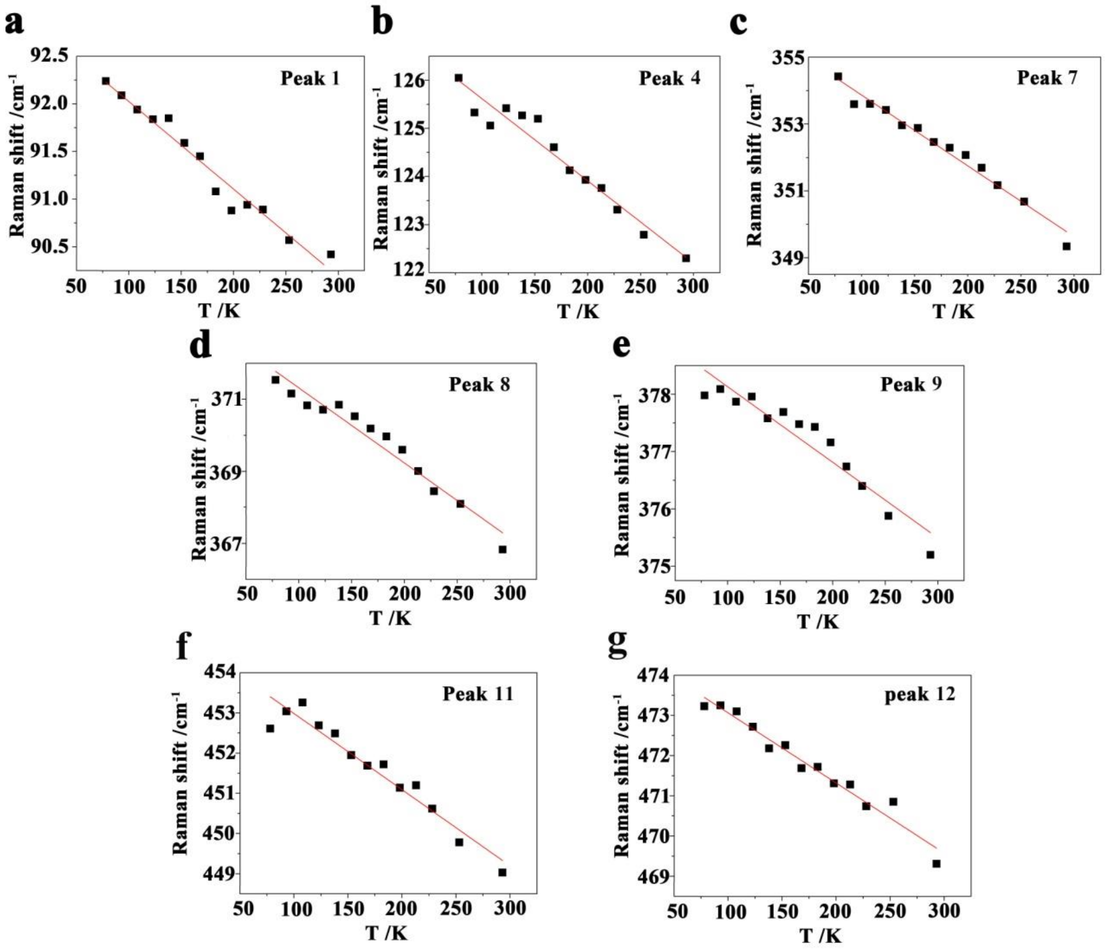

Figure S2: The peak positions of the Raman peaks of the KP_{15} nanowire at different temperatures. Temperature dependence of Raman peaks No. 1(a), 4(b), 7(c), 8(d), 9(e), 11(f), 12(g).

The frequency changes could be attributed to two reasons: the anharmonic coupling of phonons and phonon energy changes caused by thermal expansion of the lattice [4]. The measured frequency change $\triangle\omega$ can be written as:

$$\triangle \omega = (\chi_T + \chi_V) \triangle_T = \left(\frac{d\omega}{dT}\right)_V \triangle T + \left(\frac{d\omega}{dV}\right)_T \triangle V, \quad \text{(S2)}$$

where χ_T is the “self-energy” shift due to the direct coupling of the phonon modes and χ_V is the shift due to the thermal-expansion-induced volume change [5]. However, concrete contributions of “self-energy” and thermal expansion to the temperature coefficient in KP_{15} still lack theoretical research. Due to the lower thermal expansion of KP_{15} ($4.84\times10^{-6}K^{-1}$) [6], the temperature coefficient of KP_{15} nanowires is mainly due to the contribution of "self-energy" rather than to thermal expansion [4]. Therefore,

the strong temperature-dependent Raman response indicates a strong phonon–phonon coupling in KP_{15} nanowires. This result may help with non-invasive temperature measurements of KP_{15} nanodevices.

References

1. Kim, S.; Konar, A.; Hwang, W.-S.; Lee, J. H.; Lee, J.; Yang, J.; Jung, C.; Kim, H.; Yoo, J.-B.; Choi, J.-Y.; Jin, Y. W.; Lee, S. Y.; Jena, D.; Choi, W.; Kim, K. *Nature Communications.* **2012**, *3* (1), 1011.
2. Sahoo, S.; Gaur, A. P.; Ahmadi, M.; Guinel, J. F.; Katiyar, R. S. *The Journal of Physical Chemistry B.* **2013**, *117* (7), 9042-9047.
3. Late; Dattatray, J. *ACS applied materials & interfaces.* **2015**, *7* (10), 5857-5862.
4. Ca Lizo, I.; Balandin, A. A.; Bao, W.; Miao, F.; Lau, C. N. *Nano Lett.* **2007**, *7* (9), 2645.
5. Peercy, P. S.; Morosin, B. *Physical Review B.* **1973**, *7*, 2779-2786.
6. Olego, D. J. *Physical Review B Condensed Matter.* **1985**, *31* (4), 2230-2239.
